# The central signaling pathways related to metabolism-regulating hormones of the gut-brain axis: a review

**DOI:** 10.1186/s12967-025-06656-3

**Published:** 2025-06-11

**Authors:** Jiyuan Liu, Changqing Jing, Ying Guo, Zhou Shang, Baolin Zhang, Xinxiu Zhou, Jizhun Zhang, Guodong Lian, Feng Tian, Leping Li, Yuezhi Chen

**Affiliations:** 1 Department of Gastrointestinal Surgery, Shandong Provincial Hospital, Shandong First Medical University, Jinan, 250021 Shandong China; 2 Department of General Surgery, Shandong Provincial Hospital Affiliated to Shandong First Medical University, Jinan, 250021 Shandong China; 3 Department of Critical-Care Medicine, Shandong Second Provincial General Hospital, Jinan, 250021 Shandong China

**Keywords:** Obesity, Gut-brain axis, Gut-brain axis hormones, Signaling pathways, Bariatric surgery

## Abstract

Obesity is a widespread metabolic disorder linked to various conditions, including type 2 diabetes, hypertension, fatty liver disease, sleep apnea, and hyperuricemia. It significantly impacts quality of life and economic productivity. Traditional methods like diet and lifestyle changes often fail to produce substantial weight loss. Consequently, emerging treatments such as anti-obesity medications, bariatric surgery, and fecal microbiota transplantation are becoming more prominent. Recent research emphasizes the role of hormones that communicate with the hypothalamus through the gut-brain axis, affecting appetite, insulin secretion, and body weight via specific signaling pathways. This review explores the role of key gastrointestinal hormones (GLP-1, PYY, ghrelin, CCK, GIP, leptin, and bile acids) and their signaling pathways in metabolic regulation. The present research systematically evaluates the impact of bariatric surgery on appetite modulation and certain metabolic functions through key signaling pathways, including GLP-1R, GHS-R1a, and FXR/TGR5.

## Introduction

Obesity has emerged as a significant global public health challenge. The Body Mass Index (BMI) is a key metric used to classify individuals into standard weight and obesity categories. According to the World Health Organization (WHO), a BMI between 18.5 and 24.9 kg/m² is considered a standard weight, while a BMI of 30 kg/m² or higher indicates obesity [1]. As of 2022, the WHO reports that 890 million adults worldwide are diagnosed with obesity, a number that has doubled since 1990. This rise has led to an increase in health complications associated with metabolic disorders linked to obesity [1].

**Fig. 1:**
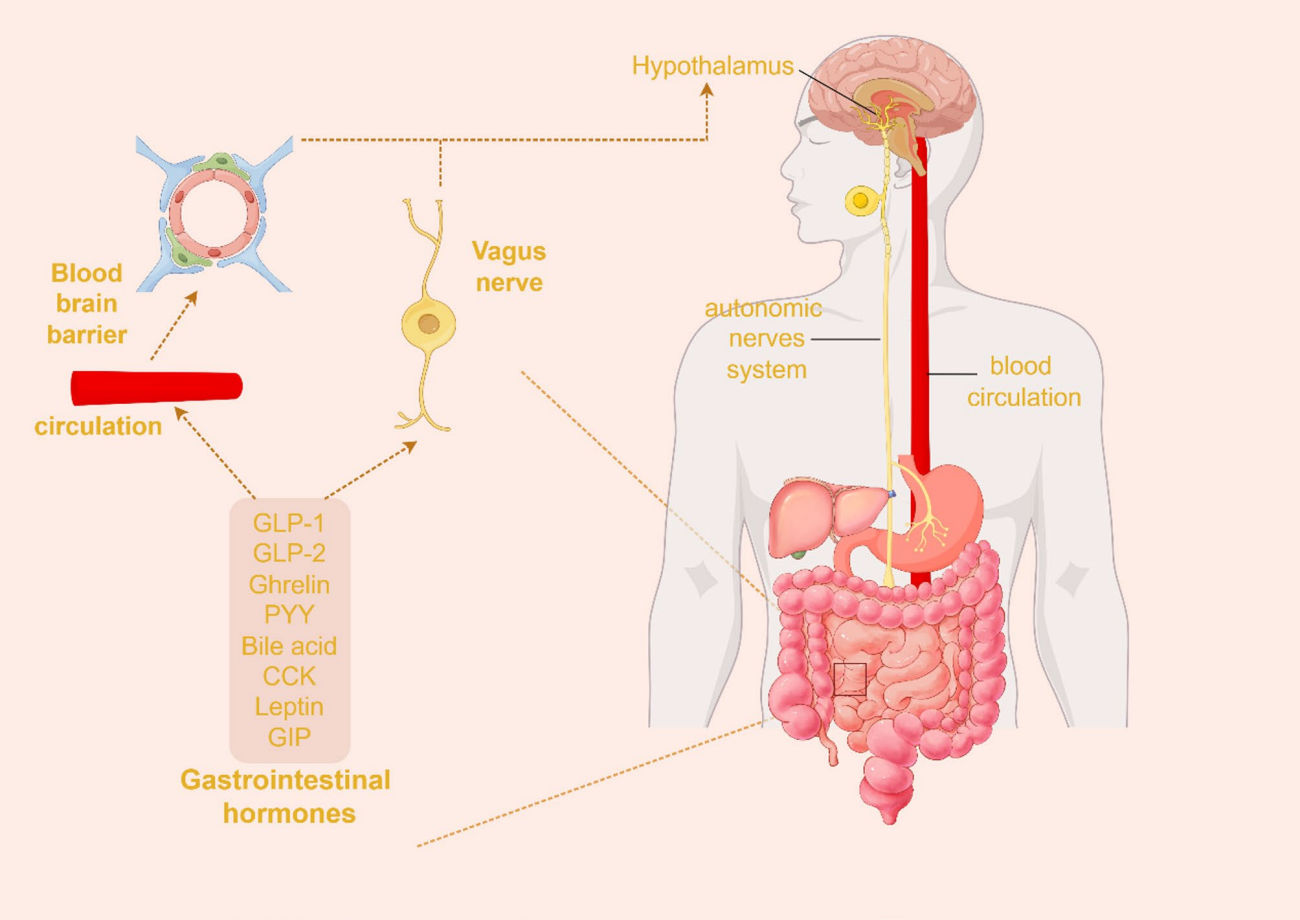
some metabolism-regulating hormones of the gut-brain axis

Body weight regulation is closely linked to the actions of multiple gastrointestinal hormones. Research shows that various hormones and cellular metabolic products regulate metabolism and weight control by accessing critical neural nuclei, such as the arcuate nucleus (ARC) via the gut-brain axis [2]. In this pathway, the vagus nerve and bloodstream are pivotal channels for transmitting signals from the gastrointestinal tract to the brain. Nutrient signals like glucose and fats, as well as hormonal signals including glucagon-like peptide-1 (GLP-1), glucagon-like peptide-2 (GLP-2), Peptide YY (PYY), ghrelin, cholecystokinin (CCK), and bile acids, enter the central nervous system (CNS) through the bloodstream or the vagus nerve. These signals exert their metabolic regulatory effects by independently or interactively transmitting through subsets of the vagus nerve or by directly crossing the blood-brain barrier into the CNS [3](Fig. 1).

Following bariatric surgery, there are changes in the expression of certain substances within the gut-brain axis neural pathways [4]. These changes can involve upregulation or downregulation of expression, which then propagate through cascade amplification effects to affect effector organs, thereby altering the body’s metabolic and endocrine states (Table 1). Compared to previous reviews, this article provides a comprehensive synthesis of recent advances in gastrointestinal hormone-related signaling pathways and elucidates how bariatric surgery influences these pathways to drive weight loss and improve metabolic outcomes. By emphasizing mechanistic insights, this review aims to inform the optimization of personalized weight-loss strategies and guide the development of non-surgical interventions that mimic the metabolic benefits of bariatric surgery [5].

**Table 1:** Postoperative changes in Gastrointestinal hormones: source and target tissues

Hormone	Origin	Receptors	Postoperative changes
GLP-1	Enteroendocrine L cell	GLP-1R	Up
GLP-2	Enteroendocrine L cell, preproglucagon neuron	GLP-2R	Up
Ghrelin	Gastric oxyntic cell	GHS-R1a	Down
PYY	Enteroendocrine L cell	Y1, Y2, Y4, Y5, and Y6 receptors	Up
Bile acids	liver parenchymal cell	FXR, TGR5	Up
CCK	Enteroendocrine L cell	CCK1R, CCK2R	Up
Leptin	Adipocyte	Leptin receptors	Down
GIP	Enteroendocrine K cell	GIPR	Up, unchanged or down

## GLP-1

### Overview

GLP-1 is a peptide composed of 36–37 amino acids, derived from the cleavage of proglucagon encoded by the Gcg gene [5]. It is predominantly produced by L cells in the terminal ileum and colon, with additional secretion occurring in the medulla and hypothalamus [5]. Once released, GLP-1 can act locally within the intestine or enter the central nervous system to regulate various physiological processes throughout the body [6].

### Central signaling

**Fig. 2:**
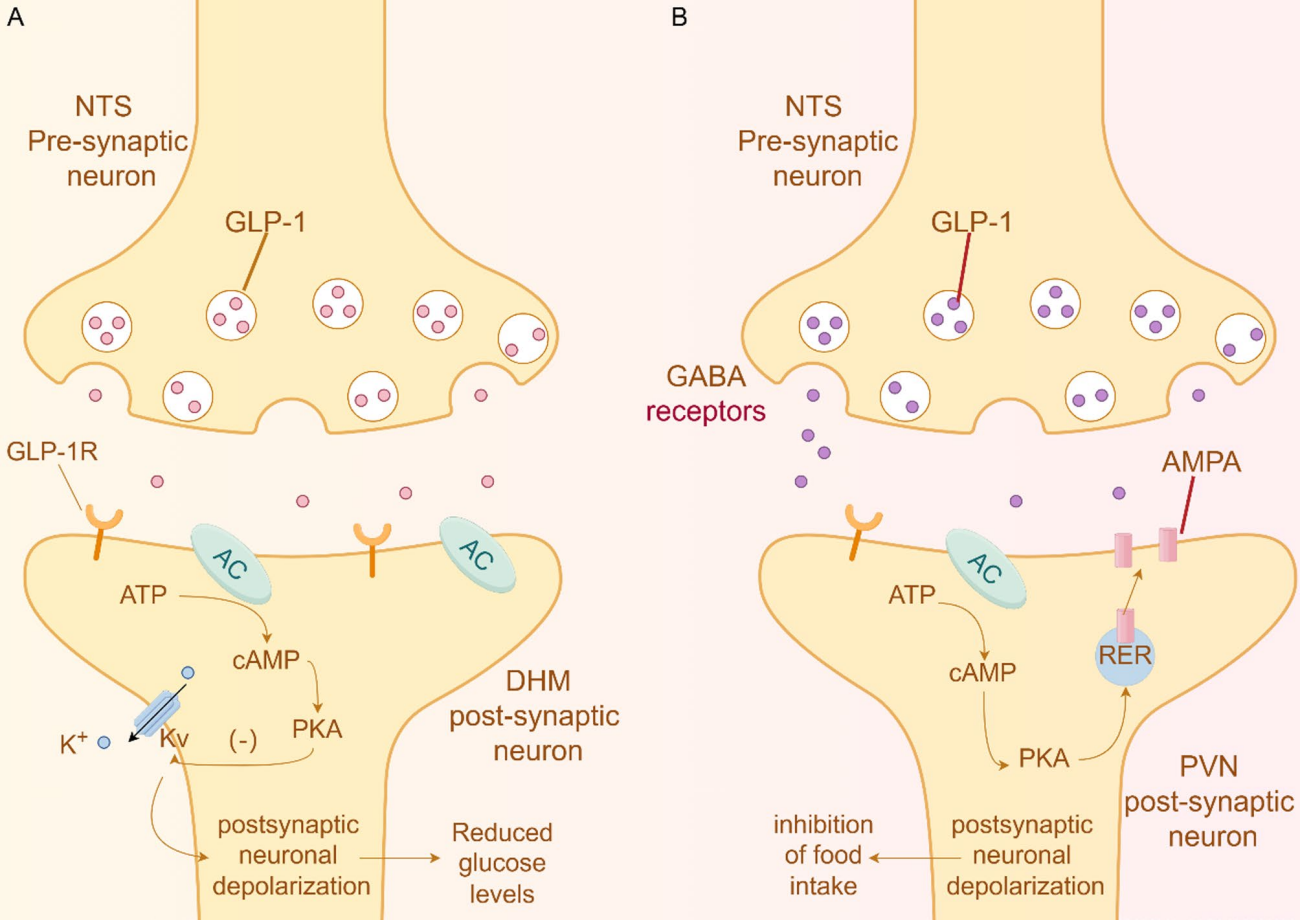
GLP-1 related signaling pathways in DHM or PVN

The gut-brain axis is the primary pathway through which GLP-1 exerts its central effects. Through this axis, GLP-1 stimulates insulin secretion, enhances insulin sensitivity, delays gastric emptying, suppresses appetite, and influences lipid metabolism [5]. Despite its significance, the exact intracellular pathways through which GLP-1 acts via the gut-brain axis remain incompletely understood due to the complexity of the neuronal mechanisms involved. Picard et al. [7] identified a subset of glucose-sensitive neurons in the dorsomedial nucleus (DMN) of the brain that express GLP-1 receptors (GLP-1R). These neurons inhibit delayed rectifier potassium channels and lower blood glucose levels through activation of the AMP-protein kinase A (cAMP-PKA) pathway (Fig. 2A). Similar glucose-sensitive neurons, categorized as glucose-excited (GE) and glucose-inhibited (GI), are found in other hypothalamic nuclei such as the ventromedial nucleus (VMN) [8]. GE neurons become more active in response to high glucose levels, whereas GI neurons exhibit the opposite response. Most GLP-1R-expressing input neurons belong to the GE type. Research indicates that GE neurons detect peripheral glucose levels through ATP-sensitive potassium channels and, by integrating signals from central GLP-1 and peripheral glucose, regulate glucose homeostasis and energy metabolism [8].

In the regulation of feeding behavior, GLP-1 neurons project to brain regions such as the ARC and the paraventricular nucleus (PVN) to exert their effects. Within the PVN, GLP-1 influences feeding behavior by modulating postsynaptic membrane excitability, likely mediated through the AC-cAMP-PKA pathway (Fig. 2B). This pathway leads to the phosphorylation of serine 845 on the GluA1 subunit of α-amino-3-hydroxy-5-methyl-4-isoxazole-propionic acid receptors (AMPARs) [9]. These receptors are predominantly found in brain regions like the hippocampus and cerebellum. Phosphorylation of GluA1 promotes the recruitment of AMPARs to the membrane, enhances excitatory postsynaptic potentials, and consequently inhibits feeding behavior [10]. In the ARC, GLP-1 has a dual effect: it activates pro-opiomelanocortin (POMC) neurons while inhibiting neuropeptide Y/agouti-related protein (NPY/AgRP) neurons. Recent in vivo electrophysiological recordings in mice indicate that both K⁺ and Ca²⁺ ions play essential roles in the anorexigenic effects of GLP-1 within the arcuate nucleus (ARC). Twelve hours after liraglutide administration, POMC neurons in the ARC exhibit depolarization and increased firing activity, an effect dependent on neuronal GLP-1 receptors and TRPC5 channels. Conversely, GLP-1 receptor agonists inhibit AgRP/NPY neurons through a mechanism involving enhanced presynaptic GABAergic input mediated by both TRPC5 and K_ATP channels, and an indirect suppression of excitatory glutamatergic input via polysynaptic circuits [11]. The alterations in membrane potential mediated by these ion channels are fundamental to the regulation of neuronal excitability and, subsequently, the control of feeding behavior. Although the activation of POMC neurons and suppression of NPY/AgRP neurons are proposed mechanisms, the exact signaling pathways remain poorly understood and require further investigation.

The gut-brain axis mechanism regulating insulin secretion and sensitivity involves the vagus nerve. GLP-1 is believed to bind to and activate vagal afferent neurons originating from the nodose ganglion, thereby increasing neuronal activity in projections to the solitary nucleus in the brainstem. These signals are then transmitted to the hypothalamus and converted into efferent vagal neurons extending to the pancreas, gastrointestinal tract, and other effector organs to regulate insulin secretion [12]. The intracellular signaling pathways implicated in GLP-1’s central regulatory effects likely include cyclic cAMP-PKA and phospholipase C-protein kinase C (PLC-PKC), among others [13]. These pathways are crucial for mediating the physiological responses to GLP-1 within the CNS, influencing insulin secretion and sensitivity.

### Postoperative changes

In healthy individuals, glucose intake stimulates increased secretion of GLP-1, playing a crucial role in maintaining glucose metabolism homeostasis. However, in rats with diet-induced obesity, GLP-1 secretion in respond to glucose stimulation is significantly impaired [14]. This impairment is alleviated by bariatric surgery. Research by Rhee et al. [15] indicates that following bariatric surgery, there is a notable increase in both GLP-1 secretion levels and blood concentrations. By the seventh day post-Roux-en-Y gastric bypass (RYGB) surgery, GLP-1 levels peak, leading to significant improvements in diabetic conditions among patients. The heightened GLP-1 levels observed after weight loss surgery may be attributed to reduced activity of dipeptidyl peptidase IV, the enzyme responsible for degrading GLP-1.

### Remaining questions

According to Elmaleh-Sachs A et al. [16], behavioral interventions typically lead to a 5–10% weight loss, while GLP-1 receptor agonists can result in an 8–21% reduction, and bariatric surgery achieves a weight loss of 25–30%. Although GLP-1 receptor agonists are somewhat less effective than bariatric surgery in terms of weight reduction, they offer a valuable alternative to standard behavioral interventions for promoting weight loss in patients with obesity. However, further research is necessary to determine the optimal dosing for both weight loss and weight maintenance, as well as the long-term dosing requirements.

## GLP-2

### Overview

GLP-2 is synthesized by enteroendocrine L cells in the intestine and by preproglucagon neurons in the brain [17]. Despite sharing a common precursor with GLP-1, GLP-2 has a distinct primary function. While GLP-2 does influence glucose metabolism to some extent, its principal role lies in promoting intestinal mucosal growth and maintaining mucosal stability [18].

### Central signaling

**Fig. 3:**
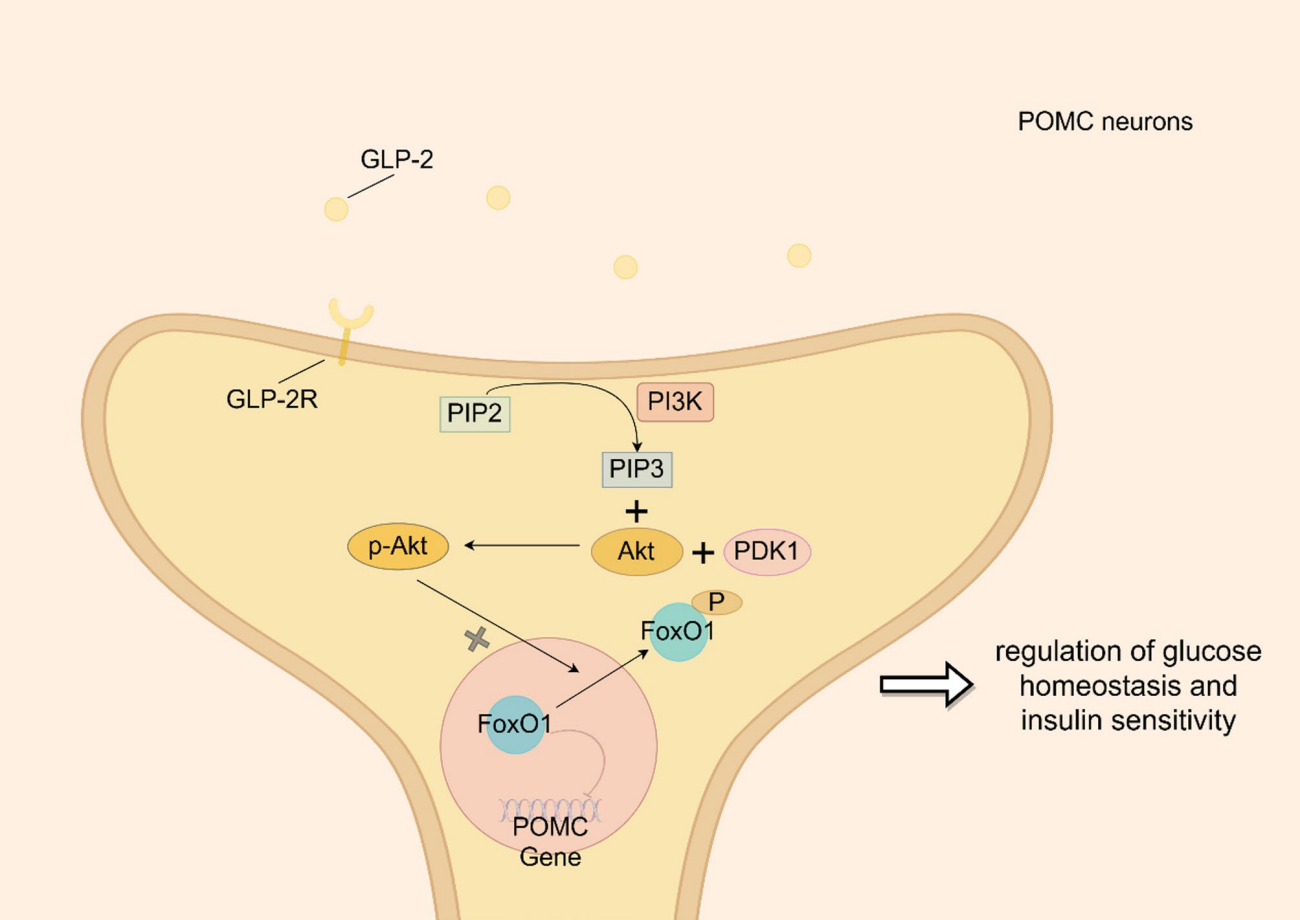
GLP-2 related signaling pathways in ARC

In the CNS, GLP-2 acts via GLP-2 receptors (GLP-2R) on POMC neurons in the hypothalamus to regulate metabolic processes [19]. The specific mechanisms by which GLP-2 modulates glucose homeostasis and insulin sensitivity in the CNS are not fully understood, but previous research suggests involvement of the GLP-2R-PI3K-Akt-FoxO1 signaling pathway (Fig. 3). In this pathway, PI3K is recruited to tyrosine residues on GLP-2R by its regulatory domain (p85) SH2 domain, leading to conformational changes in the catalytic domain (p110) and subsequent activation of PI3K. PI3K then rapidly phosphorylates Akt in primary neurons, initiating Akt-dependent inhibition of FoxO1 activity. Akt undergoes phosphorylation at Ser473 and Thr308, resulting in its active form, p-Akt, which subsequently phosphorylates FoxO1. FoxO1, a downstream target of the PI3K/Akt pathway, is phosphorylated by Akt and retained in the cytoplasm, thereby preventing its inhibition of target gene expression in the nucleus of POMC neurons [17].

In summary, GLP-2 within the CNS likely regulates membrane excitability and nuclear transcription of POMC neurons. This action suppresses hepatic glucose production and influences insulin sensitivity through activation of the PI3K-Akt-FoxO1 signaling cascade [17].

### Postoperative changes

Experimental data shows a substantial increase in GLP-2 levels following bariatric surgery. Specifically, after RYGB, GLP-2 levels rose by 91% compared to the control group (*P* = 0.02) [20]. Postprandial GLP-2 levels increased notably at 3 months post-surgery, peaked between 6 and 12 months, and gradually returned to baseline by 24 months [20]. Comparative studies among different weight loss surgery methods indicate that sleeve gastrectomy (SG) results in an even more significant elevation in GLP-2 levels, doubling those observed in the sham surgery control group [21]. Recent clinical data from Perakakis N et al. demonstrated a significant increase in GLP-2 levels following RYGB and SG procedures. After a mixed meal test, GLP-2 levels rose by approximately 2 ng/mL, exceeding the preoperative increment by over 1 ng/mL. Notably, patients who underwent RYGB exhibited a significant elevation in circulating GLP-2 levels at 3 months postoperatively compared to baseline (*p* = 0.02), with this difference remaining significant at 6 months (*p* = 0.03). In contrast, no such trend was observed in SG patients (*p* = 0.28). Given the study’s limited sample size of 28 participants, further clinical research is required to confirm these findings and elucidate postoperative hormonal changes [22]. The elevated GLP-2 levels observed post-surgery provide beneficial effects by reducing systemic inflammation and metabolic disturbances associated with obesity and diabetes [23]. Additionally, higher GLP-2 levels enhance protection of the intestinal mucosal barrier, reduce intestinal mucosal permeability, and significantly lower plasma lipopolysaccharide concentrations. These effects may be mediated by the upregulation of mRNA expression of tight junction proteins in intestinal cells [23]– [24].

## Ghrelin

### Overview

Ghrelin is a 28-amino acid protein hormone derived from the cleavage of proghrelin, which is encoded by the preproghrelin gene located on chromosome 3p26-p25 and consists of 117 amino acids [25]. It is primarily secreted by gastric oxyntic cells in the stomach, with smaller amounts synthesized in the hypothalamus, pituitary gland, and peripheral organs [26]. Ghrelin exerts its effects primarily through Growth Hormone Secretagogue Receptor 1a (GHS-R1a), a GPCR predominantly found in the pituitary gland, ARC, VMN, and PVN of the brain, as well as in various other tissues including pancreas, spleen, heart, kidneys, gonads, thyroid gland, adrenal glands, adipose tissue, and vascular systems [27]– [28]. In the CNS, ghrelin stimulates appetite by binding to GHS-R1a receptors located in brain regions such as the ARC, PVN, and DMN [29]– [30].

### Central signaling

In the VMN, ghrelin binds to GHS-R1a receptors, activates the PLC-IP3/DAG-PKC pathway, leading to a significant increase in intracellular calcium ions. Ghrelin also inhibits potassium ion channels, facilitating calcium influx through L-type calcium channels. Elevated intracellular calcium ions activate calcium/calmodulin-dependent protein kinase kinase 2 (CaMKK2), which in turn phosphorylates AMP-activated protein kinase (AMPK). The stable complex of phosphorylated AMPK with CaMKK2 enhances the phosphorylation of acetyl-CoA carboxylase (ACC) [31]. This cascade promotes the release of carnitine palmitoyltransferase 1 (CPT1) and the degradation of malonyl-CoA [26, 32]– [33]. CPT1 exists in two isoforms, CPT1A and CPT1C, with CPT1A being a rate-limiting enzyme that forms a hetero-oligomeric complex crucial for the acylcarnitine shuttle. This shuttle facilitates the translocation of long-chain fatty acids across the mitochondrial membrane for β-oxidation, thereby regulating fatty acid metabolism [34]– [35]. The hexameric structure of CPT1A interacts with acyl-CoA synthetase and voltage-dependent anion channels on the outer mitochondrial membrane, facilitating the transfer of fatty acids and enhancing β-oxidation to increase fatty acid metabolism [35].

**Fig. 4:**
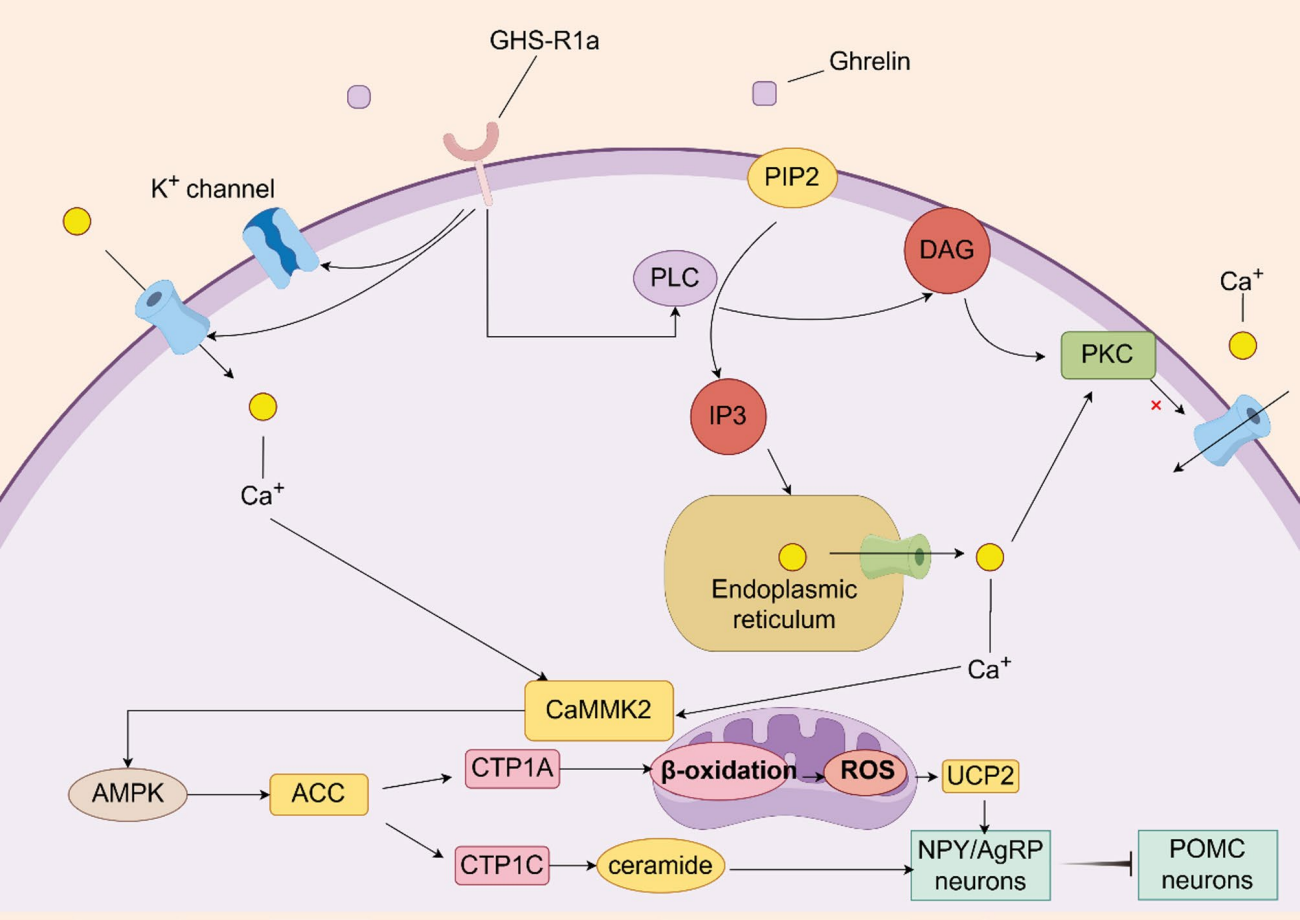
Ghrelin related signaling pathways in VMN. The pathway primarily involves the following sequence: ghrelin → GHS-R → Ca2+ → CaMKK2 → AMPK → ACC → CPT1 → (ROS → UCP2) / ceramide → NPY/AgRP neurons and POMC neurons. CTP1A is involved in mitochondrial β-oxidation, whereas CTP1C modulates appetite-regulating neurons via ceramide signaling

Increased fatty acid metabolism can lead to an upregulation of mitochondrial reactive oxygen species (ROS) production, inducing the synthesis and expression of uncoupling protein 2 (UCP2) mRNA, which is known to stimulate feeding behavior [36]. Additionally, CPT1C enhances ceramide expression and upregulates NPY and AgRP expression and secretion while downregulating POMC expression in the ARC of the hypothalamus, thereby promoting appetite. Acetyl-CoA inhibits CPT1 activity by binding to it; therefore, a decrease in acetyl-CoA levels can also promote feeding behavior. Acetyl-CoA plays a crucial role in lipid metabolism and is involved in appetite regulation, although the specific mechanisms require further investigation [37]. When ghrelin binds to GHS-R1a receptors, it initiates a cascade of reactions that ultimately regulate the activity of NPY/AgRP neurons and POMC neurons in the ARC of the hypothalamus to increase appetite. This pathway can be summarized as follows: ghrelin → GHS-R → Ca2+ → CaMKK2 → AMPK → ACC → CPT1 → (ROS → UCP2) / ceramide → NPY/AgRP neurons and POMC neurons [29](Fig. 4). However, the specific neurons within the VMN involved in these processes have not been fully elucidated.

Ghrelin increases appetite and food intake by directly acting on the ARC of the hypothalamus [38]. It activates NPY-related neurons in the PVN through the PKC-Ca2+-CaMKK2-AMPK signaling pathway, thereby promoting appetite [36, 39]. Additionally, local NPY/AgRP neurons can secrete the inhibitory neurotransmitter gamma-aminobutyric acid (GABA), which suppresses the activity of related POMC neurons, further enhancing appetite [40].

### Postoperative changes

Experimental findings indicate that following bariatric surgery, such as RYGB, ghrelin levels decrease significantly in the short term, with reductions exceeding 50% within the first two weeks. This decline contributes to improvements in glucose tolerance, promotes insulin secretion, and aids in weight reduction [41]– [42]. Researchers, including Steinert et al. [42], suggest that the rapid decrease in ghrelin secretion in the short term is primarily attributed to the rapid weight loss post-surgery and the effects of gastric partial resection on ghrelin secretion. As the body adapts over time, ghrelin secretion may gradually normalize. According to the study by Singhal V et al., one year after sleeve gastrectomy, fasting serum ghrelin levels in patients with obesity remained significantly lower, with a concentration of 36.0 ± 8.3 pg/ml, compared to 247.0 ± 35.5 pg/ml in the non-surgical control group (*p* < 0.0001) [43].A prospective cohort study conducted by Ozmen et al. demonstrated that patients with obesity who underwent sleeve gastrectomy achieved significant weight loss one year after surgery. The mean BMI decreased from 43.3 (range: 40–53) kg/m² to 29.0 (range: 23–37) kg/m². Additionally, fasting ghrelin levels exhibited a marked and sustained decline over the same period, dropping from 334.2 (range: 36.6–972.1) pg/mL to 84.0 (range: 9.1–227.0) pg/mL [44].

## PYY

### Overview

PYY is a 36-amino acid linear peptide that shares a high degree of structural similarity with NPY [45]. PYY is primarily synthesized and secreted by enteroendocrine L cells in the intestine. Its main circulating forms are PYY(1–36) and PYY(3–36) [46].

### Central signaling

**Fig. 5:**
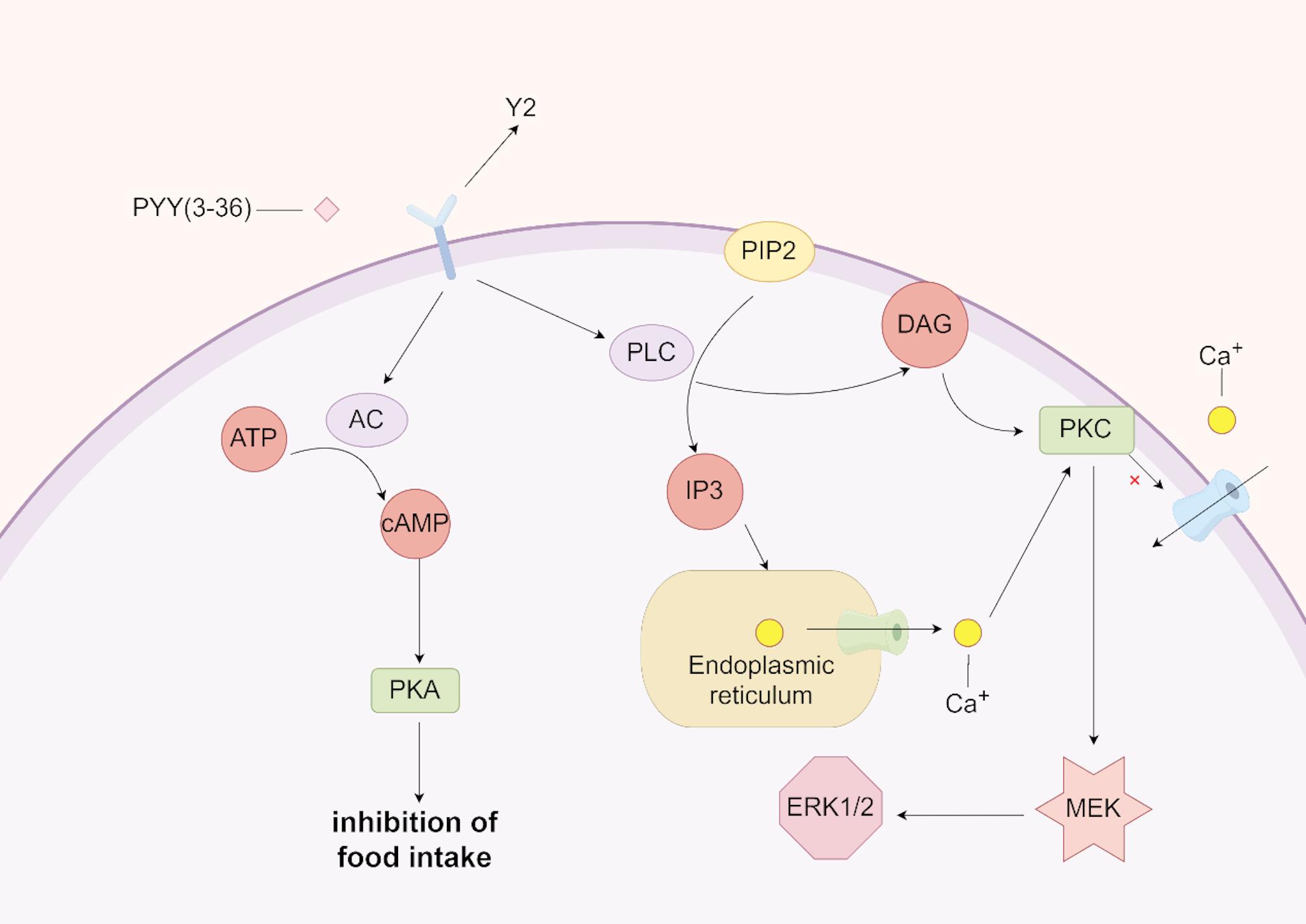
PYY related signaling pathways in ARC

PYY receptors are part of the GPCR family, primarily including Y1, Y2, Y4, Y5, and Y6 receptors [47]. Among these, the Y3 receptor is specific to NPY stimulation, while Y1 and Y2 receptors are the most predominant. The role of PYY in appetite regulation and feeding behavior is well-established [48]. When administered either peripherally with penetration through the blood-brain barrier or centrally, PYY(1–36) can induce binge eating behaviors. This effect is believed to occur through PYY(1–36) binding to Y1 receptors in the hypothalamus, which activates NPY/AgRP neurons, increases NPY expression in the ARC, and indirectly inhibits pro-opiomelanocortin/Cocaine amphetamine regulated transcript (POMC/CART) neurons [47, 49]. However, the specific neural pathways through which PYY exerts its effects are not fully understood. In contrast, PYY(3–36) binds to Y2 receptors centrally, induces conditioned taste aversion, and affects taste perception, leading to reduced food intake. PYY(3–36) crosses the blood-brain barrier from the peripheral circulation into the third ventricle, where it binds to Y2 receptors on NPY neurons in the ARC. This binding directly inhibits NPY neurons, attenuating their inhibitory effects on POMC neurons, which indirectly activates POMC neurons and decreases food intake [50]. The signaling pathways mediated by PYY(3–36) binding to Y2 receptors in the ARC may involve several mechanisms. PYY(3–36) inhibits adenylate cyclase (AC) activity through Giα coupling, thereby suppressing the cAMP-PKA signaling pathway. Inhibition of AC results in decreased intracellular cAMP levels, thereby reducing PKA activity. Additionally, upon coupling with the Y₂ receptor, Gαq activates phospholipase Cβ (PLCβ), a key enzyme that catalyzes the hydrolysis of the membrane phospholipid phosphatidylinositol-4,5-bisphosphate (PIP₂). In addition to Gαq coupling, the Y₂ receptor can also activate PLC through the βγ subunits of Gi. The Giβγ dimer facilitates the recruitment of PLCβ to the plasma membrane, thereby promoting its activation. PLC mediates the conversion of PIP₂ into membrane-bound diacylglycerol (DAG) and soluble inositol 1,4,5-trisphosphate (IP₃). It is currently understood that PLCβ increases intracellular Ca²⁺ concentrations via PIP₂ hydrolysis, a process co-regulated by Gβγ dimers released from both Gαq and Gi proteins. The resulting elevation in intracellular Ca²⁺ activates protein kinase C (PKC), which subsequently phosphorylates and activates MEK, ultimately leading to enhanced phosphorylation and activation of ERK1/2 within the MAPK pathway [51] – [52](Fig. 5).

### Postoperative changes

After bariatric surgery, circulating levels of PYY show a sustained increase, which correlates with reduced food intake and significant alleviation of obesity [53]. This effect is particularly notable in patients with obesity and those without diabetes [54]. A meta-analysis by Simoneau M et al. on hormone changes before and after RYGB in patients with obesity showed that, at 26 and 52 weeks postoperatively, four studies reported fasting total PYY levels of 116 pg/ml (95% CI: 107–125) and 123 pg/ml (95% CI: 104–142), respectively. These postoperative levels were significantly higher compared to preoperative values (*p* = 0.034 and 0.030) [55]. Clinical data from Dimitriadis E et al. demonstrated a significant increase in postprandial PYY levels after sleeve gastrectomy compared to preoperative levels. At 6 months postoperatively, postprandial PYY levels at 60 min were 79.56 (43.43–129.7) pg/ml (*p* = 0.018), and at 120 min, 108.65 (37.54–140) pg/ml (*p* = 0.02). At 12 months postoperatively, the postprandial PYY levels at 60 min were 74.75 (49.62–109.38) pg/ml (*p* = 0.008), and at 120 min, 99.6 (20.31–158.78) pg/ml (*p* = 0.011) [56].

### Remaining questions

Unlike GLP-1, the elevated levels of PYY post-surgery may contribute to longer-term improvements in obesity and glucose tolerance, highlighting its substantial and lasting role in weight reduction compared to GLP-1 [57]. However, the precise mechanisms underlying the increase in PYY after surgery are still not fully understood.

## Bile acids

### Overview

Bile acids are primarily synthesized from cholesterol in the liver through both the classic and alternative pathways, with the classic pathway being the predominant route [58]. Recent research indicates that bile acids play a significant role in improving overall metabolism and promoting weight loss following bariatric surgery by activating signaling pathways involving the farnesoid X receptor (FXR) and the G protein-coupled bile acid receptor (TGR5) [59]. FXR is widely distributed across various organs, where it binds bile acids and directly enhances insulin secretion in pancreatic β-cells [60]. In the intestines, activation of FXR also regulates glucose and lipid metabolism [61]– [62]. Additionally, intestinal FXR activation leads to the upregulation of fibroblast growth factor 19 (FGF19), a key player in metabolism [63].

### Central signaling

**Fig. 6:**
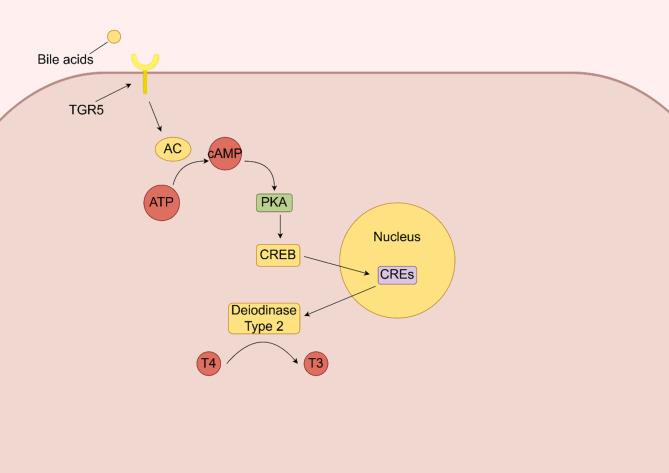
Bile acids related signaling pathways in brown adipose tissue and muscle

**Fig. 7:**
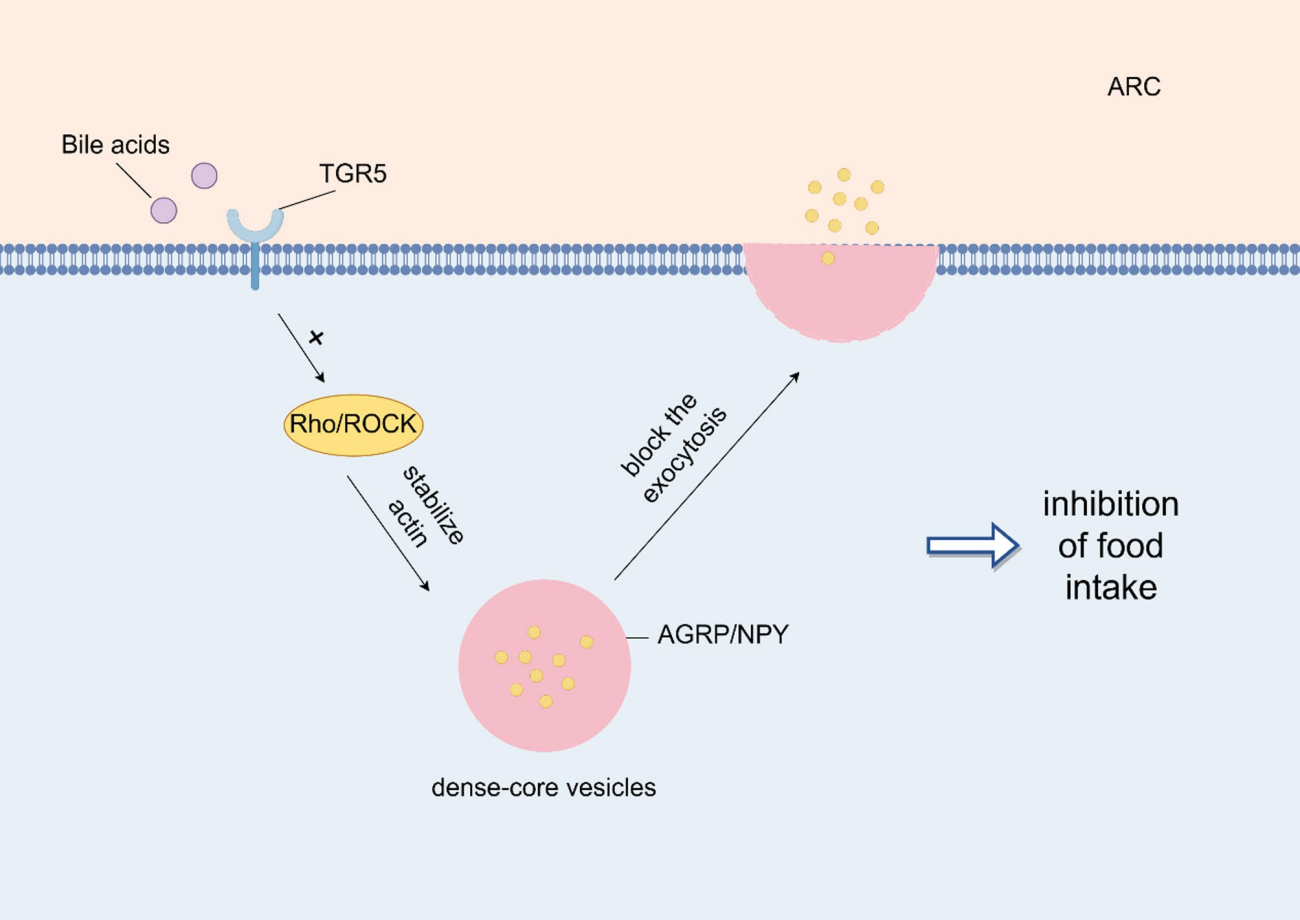
Bile acids related signaling pathways in ARC

In the CNS, FGF19 helps inhibit food intake, reduce blood glucose levels, and increase energy expenditure, thus linking the bile acid gut-brain axis metabolic signaling pathway [64]. While the exact CNS signaling pathways of FGF19 are not fully elucidated; however, they may involve the activation of molecules such as FRS1/2, ERK1/2, and STAT3 [65]. These pathways contribute to the regulation of food intake, glucose metabolism, and energy balance through the CNS-mediated effects of bile acids and their downstream signaling molecules. TGR5, a GPCR widely distributed in organs such as the intestine, pancreas, and skeletal muscle [66], utilizes the classical cAMP-PKA signaling pathway to regulate several essential functions (Fig. 6). In intestinal L cells, activation of TGR5 promotes the secretion of GLP-1, which is crucial for regulating glucose metabolism and insulin secretion [67]. Concurrently, TGR5 enhances thyroid hormone production in brown adipose tissue and muscle, thereby increasing the metabolic rate, while also exhibiting hepatoprotective effects that benefit liver health [67]. In the embryonic mouse hypothalamic cell line mHypoE-N41, bile acids stimulate TGR5 via the Rho/Rho-associated protein kinase pathway (Fig. 7), effectively inhibiting neuropeptide release from NPY/AgRP neurons, which suppresses appetite。In mHypoE-N41 cells, TGR5 activation enhances the phosphorylation of ROCK classic targets, including myosin light chain (MLC) and COFILIN. Additionally, bile acids (BAs) induce COFILIN phosphorylation, suggesting that BAs activate the ROCK signaling pathway. However, the study does not directly report the in vivo activation of the ROCK pathway, such as the phosphorylation status of MLC or COFILIN in hypothalamic tissue. In conclusion, the current data support the involvement of Rho/ROCK in TGR5-mediated regulation of AgRP/NPY secretion at the cellular level. Yet, whether Rho/ROCK directly mediates TGR5 effects in vivo remains to be confirmed by additional experiments, such as in vivo gene knockout models and Western blot analysis of hypothalamic tissue [68]. Additionally, TGR5 activation in the nodose ganglia triggers POMC and CART neurons, further reducing food intake and appetite signals [69]. Overall, TGR5 plays a pivotal role in regulating GLP-1 secretion, metabolism, hepatoprotection, and appetite suppression through distinct signaling pathways across various tissues and organs.

### Postoperative changes

Following bariatric surgery, there is a significant increase in total bile acids, which exert metabolic effects through receptors such as TGR5, impacting both the gut-brain axis and the peripherally system [70]. Recent data demonstrate that acute central administration of 5 µg of the centrally selective TGR5 agonist, 3-(2-chlorophenyl)-N-(4-chlorophenyl)-N,5-dimethyl-4-isoxazolecarboxamide, significantly reduces body weight gain in mice. Compared with vehicle-treated controls, mice receiving the agonist exhibited over 1 g less weight gain within 24 h. Furthermore, chronic central administration of 5 µg/day for 4 weeks resulted in a significant reduction in body weight from baseline, with treated mice weighing approximately 10 g less than controls at the end of the treatment period (*p* < 0.01) [71]. Makki et al. demonstrated that hyodeoxycholic acid, a potent TGR5 agonist, significantly attenuated weight gain in mice. Oral gavage of 50 mg/kg hyodeoxycholic acid three times per week for 6 weeks resulted in approximately 10% less body weight gain relative to baseline compared with vehicle-treated controls (*p* < 0.001) [72].

## CCK

### Overview

CCK is a peptide hormone primarily secreted by enteroendocrine cells in the intestine, with higher concentrations in the gastrointestinal tract and the central nervous system [73]– [74]. CCK has diverse physiological functions, including stimulating gallbladder contraction, increasing pancreatic enzyme secretion, delaying gastric emptying, and enhancing insulin release [75]– [76]. Beyond its digestive roles, CCK also regulates appetite and may influence emotions, circadian rhythms, and memory [75, 77–79]. Elevated levels of CCK promote feelings of satiety, suppress food intake, and reduce overall energy consumption [80]. CCK receptors, classified as GPCRs, consist of CCK1R and CCK2R, which are widely distributed throughout the body and play a crucial in gut-brain axis signaling [81]. In the intestine, CCK acts on vagal afferent nerves (VANs), regulating its own signals and integrating inputs from other substances acting on VANs. These integrated signals are transmitted to the solitary nucleus to modulate gastric emptying and food intake [82]– [83].

### Central signaling

Studies have shown that CCK injection results in an upregulation of c-Fos expression in the NTS, providing strong evidence for its activation. Additionally, the study observed activation in appetite-related brain regions such as the NTS, PVN, and ARC following combined administration of the GLP-1 receptor agonist AC3174 and the CCK1R receptor agonist AC170222. However, this neuronal activation was abolished upon treatment with the CCK1R antagonist (e.g., Lorglumide), indicating that CCK1R plays a critical role in the signaling pathway [84]. Currently, the specific second messengers involved in the CCK signaling pathway from the NTS to hypothalamic regions remain unclear. However, studies on the combined treatment of AC3174 and AC170222 for obesity have shown an upregulation of calcitonin receptor-like (Calcrl) gene expression. RNAscope analysis revealed that Calcrl⁺ neurons are primarily located at the junction of the NTS and the area postrema (AP). Furthermore, under combined treatment, 85% of activated neurons in the AP and 50% of activated neurons in the NTS expressed Calcrl. This suggests that Calcrl⁺ neurons play a crucial role in the excitatory response of the medulla oblongata to combined agonists, potentially providing new insights into the second messenger pathways that mediate CCK signaling from the medulla oblongata to the hypothalamus [85].

### Postoperative changes

Following bariatric surgery, particularly procedures like SG and RYGB, there is a notable increase in serum CCK levels, which correlates with enhanced activity in CCK-related metabolic pathways. However, the precise mechanisms behind these changes remain unclear. Proposed hypotheses include increased proliferation of intestinal CCK-secreting cells, heightened parasympathetic nerve activity, and activation of factors that promote CCK release within the intestinal lumen [42, 53, 86, 87]. Further research is needed to fully elucidate these mechanisms and their implications for post-surgical metabolic adaptations. After bariatric surgery, the secretion of CCK increases significantly, contributing to the suppression of liquid gastric emptying. Additionally, CCK exerts central effects through the vago-vagal reflex by increasing pyloric sphincter pressure and reducing motility in the antrum and duodenum, thereby prolonging gastric retention. These mechanisms collectively enhance feelings of satiety and extend the duration of digestion [42].

## Leptin

### Overview

Leptin is a peptide hormone primarily produced by adipose tissue, though it is also expressed to some extent in organs such as the gastrointestinal tract and brain [88]. Circulating leptin levels are positively correlated with body fat mass and short-term energy intake, making it a useful biomarker for assessing both adiposity and short-term energy balance [89]– [90]. Leptin exerts its effects on various regions of the hypothalamus, including the ARC, VMH, DMN, and lateral hypothalamus (LH), with the ARC being a principal site of action [91]. Functionally, leptin regulates energy balance and glucose homeostasis primarily by modulating the electrical activity of neurons, particularly influencing AgRP and POMC neurons in the ARC [92].

### Central signaling

**Fig. 8:**
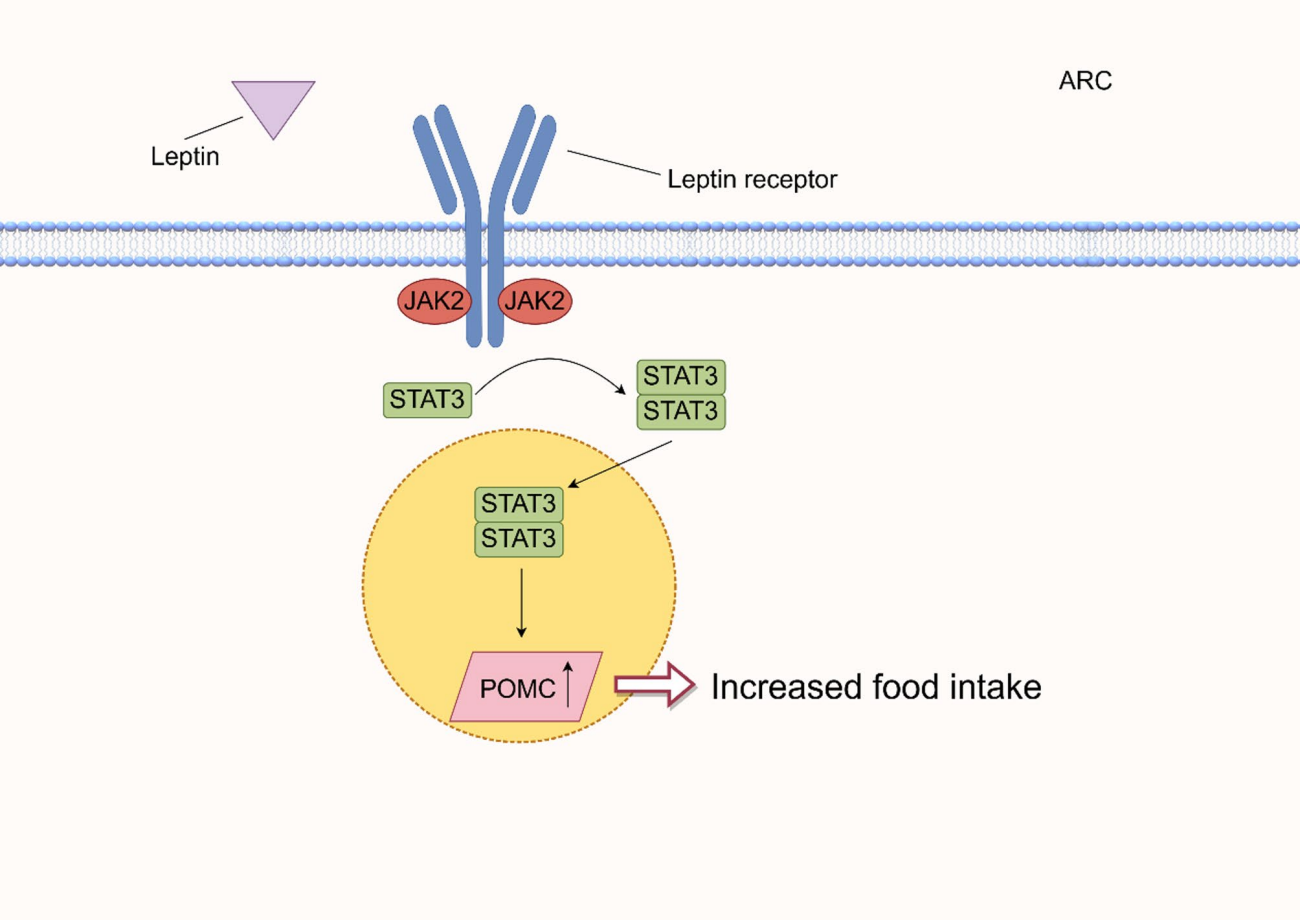
Leptin primarily targets POMC-associated genes and also modulates the expression of AGRP and NPY genes, together regulating food intake

Through activation of the Janus kinase 2 (JAK2)/signal transducer and activator of transcription 3 (STAT3) pathway, leptin exerts its regulatory influence on the ARC of the hypothalamus. This pathway begins with leptin binding to its receptor, leading to the activation of JAK2. JAK2 then phosphorylates tyrosine residues on the leptin receptor, creating docking sites for STAT3. Once STAT3 is recruited and phosphorylated by JAK2, it forms dimers that translocate to the nucleus where they act as transcription factors. In the ARC, activation of the JAK2/STAT3 pathway by leptin inhibits the activity of neurons expressing NPY and AgRP, which are orexigenic (appetite-stimulating). Simultaneously, it activates POMC neurons, which produceα-MSH. α-MSH then binds to melanocortin receptors (MCR3 and MCR4) in the PVN, suppressing appetite and promoting energy expenditure. Thus, the JAK2/STAT3 pathway (Fig. 8) serves as the primary signaling mechanism through which leptin regulates energy balance in the hypothalamus, playing a crucial role in maintaining body weight and glucose homeostasis [93]– [94].

Additionally, leptin exerts influence over the expression of substances like dynorphin and brain-derived neurotrophic factor, both directly and indirectly through neuronal projections originating from the ARC and other hypothalamic regions. These effects are integral to mechanisms involved in weight loss.

### Postoperative changes

In patients with obesity, elevated circulating leptin levels can result in excessive stimulation of leptin receptors, leading to receptor desensitization and impaired signaling, contributing to the dysregulation of energy balance seen in obesity. This phenomenon, often referred to as “leptin resistance,” plays a significant role in the pathophysiology of obesity, where despite high levels of leptin, the CNS fails to adequately respond, leading to continued overeating and reduced energy expenditure [95]. However, following weight loss surgery, evidence suggests that the CNS becomes more sensitive to leptin. Stefater MA et al. reported that by postoperative day 50, plasma leptin levels in rats undergoing SG were reduced by at least 5 ng/mL compared to sham-operated controls (*p* < 0.001). Notably, after three consecutive days of intraperitoneal administration of leptin, SG-treated rats exhibited a significantly greater suppression of food intake than sham-operated rats, indicating enhanced leptin sensitivity following surgery [96]. A recent study by Chen et al. demonstrated that the suppression of hypothalamic toll-like receptor 4 (TLR4) inflammatory signaling and the alleviation of endoplasmic reticulum (ER) stress are closely associated with the restoration of central leptin sensitivity. In rats undergoing Roux-en-Y gastric bypass (RYGB), transcript levels of Tlr4, Myd88, Cd14, and downstream pro-inflammatory cytokines including Il1b, Il6, Il18, and Tnfa were significantly reduced in the hypothalamus, suggesting a procedure-specific attenuation of TLR4 signaling. Central administration of the TLR4 inhibitor TAK242 enhanced leptin-induced suppression of food intake in rats with diet-induced obesity, but did not exert additive effects in RYGB-treated animals, indicating that RYGB may exert its benefits through this mechanism. In contrast, induction of ER stress via central administration of thapsigargin completely abolished the RYGB-mediated improvement in leptin sensitivity, without affecting control animals, highlighting ER stress as a key regulatory factor in the beneficial effects of RYGB on hypothalamic leptin signaling [97]. After biliopancreatic diversion (BPD), women with obesity exhibit a marked improvement in insulin sensitivity, which appears to be closely associated with changes in leptin signaling. Previous studies have demonstrated that leptin dose-dependently inhibits glucose-stimulated insulin secretion in isolated pancreatic β-cells, and significantly reduces insulin levels in perfused pancreatic preparations. Following BPD, the 24-hour mean circulating leptin concentration decreased from 51.94 ± 9.21 to 15.04 ± 4.80 ng/ml, while insulin levels dropped from 54.84 ± 6.51 to 10.03 ± 1.48 ng/ml. Although this observation appears inconsistent with leptin’s inhibitory effect on insulin secretion, it can be explained by the restoration of leptin sensitivity after surgery. Prior to BPD, patients typically present with hyperleptinemia-induced central leptin resistance. Postoperatively, despite reduced circulating leptin levels, sensitivity to leptin is restored, enabling it to exert its physiological inhibitory effect on insulin secretion. This restoration helps reestablish both the circadian and dynamic rhythm of insulin release, leading to a more than twofold increase in insulin sensitivity, as measured by euglycemic clamp, compared with preoperative values [98].

## GIP

### Overview

Gastric inhibitory polypeptide (GIP) is a 42-amino acid peptide hormone produced by enteroendocrine K cells located in the duodenum and jejunum of the gastrointestinal tract. After food intake, circulating levels of GIP typically increase. The GIP receptor (GIPR), a GPCR, is primarily expressed in pancreatic β-cells, where it plays a key role in insulin secretion. Additionally, GIPR is found at lower expression levels in adipose tissue and the CNS [99].

### Central signaling

Research indicates that GIP’s effects on weight reduction and appetite suppression primarily occur through the signaling of GIPR in the CNS. Conversely, GIP may also promote weight gain by interacting with receptors in peripheral adipose tissue [100]. Interestingly, studies have shown that administration of GIP, whether delivered peripherally or centrally, can significantly decrease both body weight and food intake [99]. In the periphery, both in vivo and in vitro studies have demonstrated that GIP binding to its receptor (GIPR) increases intracellular cAMP levels in pancreatic β-cells, thereby activating PKA. PKA, in turn, phosphorylates the Kir6.2 and SUR1 subunits of ATP-sensitive potassium (KATP) channels, reducing their activity. It also phosphorylates the α1 and β2 subunits of L-type voltage-dependent calcium (VDC) channels, promoting their activation. The resulting membrane depolarization due to KATP inhibition, together with direct activation of VDC channels, facilitates calcium influx and promotes insulin granule exocytosis.

Additionally, GIP-mediated cAMP elevation activates exchange protein directly activated by cAMP 2 (Epac2), which stimulates ryanodine receptors (RYRs) on the endoplasmic reticulum to release stored calcium, further increasing cytosolic Ca²⁺ concentration. Notably, the insulinotropic effect of the Epac2 pathway is only significant when glucose concentrations are at or above 5.5 mM, ensuring that GIP stimulates insulin secretion primarily under hyperglycemic conditions.

Interestingly, GIP also promotes glucagon secretion. In vitro studies have shown that at glucose concentrations below 5.5 mM, GIP activates the cAMP/PKA pathway, enhancing calcium influx and triggering exocytosis of glucagon.

In lipid metabolism, GIP promotes triglyceride storage under hyperinsulinemic conditions, but stimulates lipolysis and fatty acid oxidation under normal or low insulin levels. Studies indicate that in the presence of insulin, GIP enhances phosphorylation of protein kinase B (PKB/Akt) and inhibits phosphorylation of LKB1 and AMPK, leading to activation of lipoprotein lipase (LPL) and lipid accumulation. GIP also facilitates nuclear translocation of the CREB-regulated transcription coactivator 2 (TORC2), which binds to the LPL promoter to induce its expression. Furthermore, GIP promotes GLUT4 translocation in 3T3-L1 adipocytes, enhancing insulin-stimulated glucose uptake and its conversion into lipids.

These peripheral effects, while independent of appetite regulation, contribute to energy storage and body weight control.

Centrally, GIP acts on Vgat-positive inhibitory GABAergic neurons to induce c-Fos expression and modulate hypothalamic feeding circuits. It is proposed that GIP transmits satiety signals via neural projections from the area postrema (AP) to the nucleus tractus solitarius (NTS), parabrachial nucleus, and central amygdala. While the exact mechanisms remain unclear, these pathways are thought to play a role in appetite regulation and energy balance. Moreover, GIPR is sparsely expressed in POMC neurons in the ARC and peripheral administration of acyl-GIP has been shown to activate these neurons, thereby suppressing appetite through the melanocortin system [101].

The weight-reducing effects of centrally administered GIP are primarily mediated through interactions with GABAergic neurons in regions such as the ARC of the hypothalamus. These interactions are vital for regulating feeding behavior and energy balance. In contrast, peripheral administration of GIP may induce mechanisms that lead to GIP-mediated resistance to leptin within brain cells. This resistance occurs through the cAMP-EPAC-RAP1 signaling pathway, which significantly reduces leptin-induced appetite suppression and subsequent weight loss [100–103]. However, while these mechanisms have been proposed, the specific brain regions where GIP exerts its effects and the precise neural pathways involved remain under investigation. Future studies are needed to clarify the detailed mechanisms by which GIP influences appetite, weight regulation, and metabolic processes in both central and peripheral contexts.

### Postoperative changes

Following bariatric surgery, changes in circulating GIP levels show procedure-specific variability. Studies indicate that SG often leads to increased postprandial GIP levels, although this increase is not consistently in all patients. Perakakis N et al. reported that circulating GIP levels increased by more than 200 pg/mL following a mixed meal test (MMT) in individuals with obesity after sleeve gastrectomy (SG), while fasting GIP levels remained largely unchanged [22]. Similarly, a meta-analysis by McCarty TR et al. confirmed that SG had no significant effect on fasting GIP levels (Hedges’s g = -0.213, 95% CI: -1.019 to 0.592, *p* = 0.604) [104]. This elevation in GIP contributes to weight loss and improved glycemic control in SG patients [105]. Conversely, RYGB surgery alters nutrient transit by bypassing the duodenum and jejunum, which can affect postprandial GIP secretion from mucosal K cells. This surgical modification results in varied outcomes, with post-surgical GIP levels either decreasing, remaining stable, or increasing. Therefore, the metabolic benefits associated with RYGB are not uniformly significant [22]. A meta-analysis by Gao Z et al. demonstrated a significant reduction in fasting GIP levels after Roux-en-Y gastric bypass (RYGB) in individuals with obesity (SMD = 0.38, 95% CI: 0.21 to 0.56, *p* < 0.0001) [106]. However, Perakakis N et al. observed no significant change in fasting GIP levels following RYGB, but reported a marked decrease in postprandial GIP levels at 3 months post-surgery (*p* = 0.007), which persisted at 6 months (*p* = 0.002) and 12 months (*p* < 0.001) [22]. Understanding these nuances is crucial for optimizing clinical outcomes and managing metabolic health following different bariatric surgery procedures. Further research is needed to clarify the mechanisms underlying divergent GIP responses post-surgery (Table 2).

Changes in gut-brain axis hormone production may be attributed to alterations in nutrient delivery pathways (such as the bypass-induced rapid transport of nutrients), redirection of bile flow, and modifications in the gut microbiota. Both sleeve gastrectomy (SG) and Roux-en-Y gastric bypass (RYGB) create direct or indirect functional bypasses that accelerate nutrient delivery. Postoperatively, structural remodeling of the gut enhances the transport rate of substances, such as bile acids, within the small intestine, thereby increasing their circulating concentrations. This remodeling also alters the directional flow of bile and other substances, causing them to bypass certain segments of the small intestine, which impacts their reabsorption and circulation. Furthermore, postoperative changes in gut microbiota diversity, spatial organization, and stability may contribute to shifts in gut-brain axis hormone levels. For example, certain gut microbiota produce short-chain fatty acids, which stimulate the secretion of GLP-1 via free fatty acid receptor-2 [61, 107].

**Table 2:** Known Gut-Brain Axis hormones and their association with bariatric surgery

Hormone	Postoperative Changes	Signaling Pathways
**GLP-1**	↑	GLP-1R → AC → cAMP → PKA →Kv/AMPK
**GLP-2**	↑	GLP-2R → PI3K → Akt → FoxO1
**Ghrelin**	↓	GHS-R → AMPK → ACC→ (ROS → UCP2) / ceramide
**PYY**	↑	Y2 → AC → cAMP → PKA Y2 → PLC → PKC → ERK1/2
**Bile acids**	↑	TGR5 → AC → cAMP → PKA TGR5 → Rho/ROCK
**CCK**	↑	CCK1R → Calcrl^a^
**Leptin**	↓	leptin receptor → JAK2 → STAT3
**GIP**	↑, ↔ or ↓	GIPR → cAMP → PKA/Epac2^a^

For more detailed information, please refer to the complete text of the article. ^a^, currently, the specific signaling mechanisms by which CCK and GIP exert their metabolic benefits after bariatric surgery in the CNS remain unclear. GLP-1, glucagon-like peptide-1; GLP-2, glucagon-like peptide-2; PYY, peptide YY; CCK, cholecystokinin; GIP, gastric inhibitory polypeptide.

**Fig. 9:**
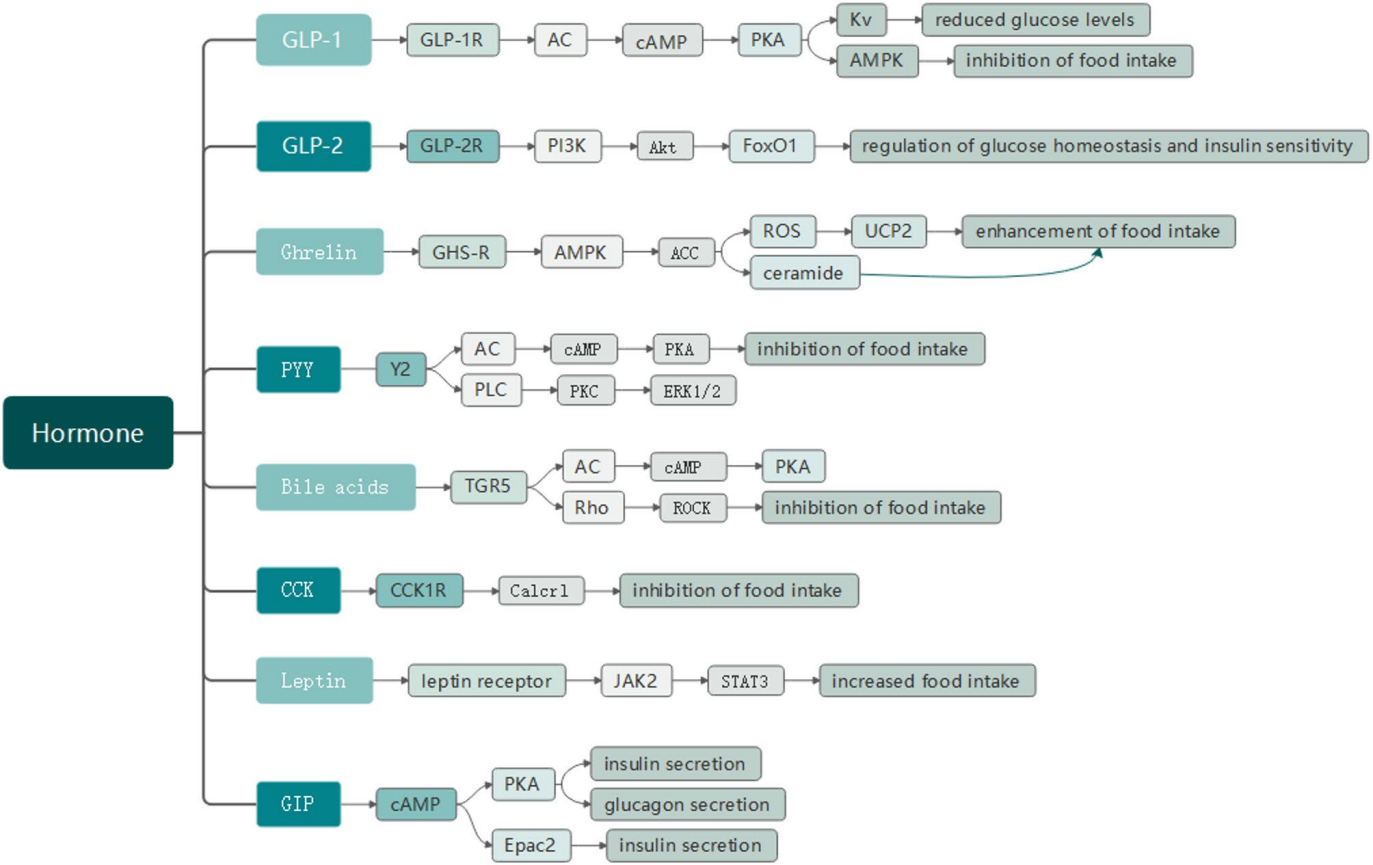
A set of flowcharts summarizing key gut-brain axis hormones, their receptors, primary intracellular signaling pathways, and associated physiological effects

## Conclusion

Building upon the physiological roles of gut-brain axis hormones and their modulation by bariatric surgery, this review places greater emphasis on the underlying signaling mechanisms, particularly the intracellular pathways activated by these hormones in both central and peripheral tissues. Moreover, we adopt an interdisciplinary perspective that integrates neuroendocrinology and metabolic physiology, offering a synthesis that is less commonly addressed in existing reviews.

Following bariatric surgery, significant increases in GLP-1, GLP-2, PYY, bile acids, and CCK levels were observed, which contribute to appetite suppression and weight loss. In contrast, leptin and ghrelin levels decreased markedly, and GIP also exhibited coordinated changes. Collectively, these hormonal alterations contribute to improved metabolic outcomes post-surgery (Fig. 9).

Bariatric surgery patients and animal models have demonstrated that hormones involved in the gut-brain axis influence metabolism. The surgery, which restricts dietary intake, works synergistically with alterations in gut-brain axis hormone levels to enhance metabolic outcomes. Notably, the emerging GLP-1 receptor agonists have shown weight loss efficacy that may even exceed that of bariatric surgery. As research continues to evolve, the complex relationship between gut-brain axis hormones and overall organismal metabolism is becoming increasingly evident. Gut-brain axis hormones transmit metabolic regulatory signals from the intestine to specific brain regions through the circulatory or nervous systems, thereby modulating whole-body metabolism. Understanding the specific signaling pathways by which these hormones affect metabolic processes is essential for enhancing our comprehension of body weight and energy balance regulation. This knowledge serves as a basis for investigating whether pharmacological or other interventions can replicate the changes in gut-brain axis hormones observed after bariatric surgery, aiming to achieve metabolic benefits while minimizing surgical risks. In the multicenter SURMOUNT trial, a 72-week treatment with tirzepatide, a dual GLP-1R/GIPR agonist, resulted in a 20.9% reduction in body weight—17.7% greater than that observed in the placebo group. Typically, bariatric surgery achieves a 25–30% weight loss within 1–2 years. By exerting both central and peripheral metabolic regulatory effects, tirzepatide has substantially narrowed the gap between pharmacological and surgical weight loss, representing a significant milestone in the advancement of multi-receptor agonist therapies for obesity [108]. Bossart et al. demonstrated that Macaca fascicularis with diet-induced obesity treated with the GLP-1/GIP/GCGR triagonist SAR441255 for 42 days experienced a significant body weight reduction of 12.6% ± 1.74% compared to controls (*p* < 0.05). In comparison, animals receiving a GLP-1/GCGR dual agonist showed an 8.1% ± 1.7% decrease (*p* < 0.05). This superior weight loss achieved with the triagonist supports the continued development of multi-receptor agonists [109]. Similarly, Retatrutide induced a dose-dependent reduction in body weight. After 36 weeks, participants in the 12 mg group exhibited a mean weight loss of 16.94%, which was significantly greater than that observed with placebo (3.00%) or dulaglutide (2.02%) (*p* < 0.0001). Notably, 71–75% of individuals in the 8 mg and 12 mg groups lost more than 10% of their body weight, while reductions of over 15% and 20% were achieved by 57–63% and 39–40% of participants, respectively [110].

While considerable progress has been made in elucidating the mechanisms of gut-brain axis hormones such as GLP-1 and ghrelin in both central and peripheral tissues following bariatric surgery, the intracellular signaling pathways of other hormones remain insufficiently understood. For instance, the specific neuronal populations and second messenger systems through which CCK and GIP regulate appetite and body weight in the hypothalamus have yet to be clearly defined. This uneven progress reflects the varying maturity of different research areas rather than differences in physiological importance. Future investigations into receptor coupling mechanisms, second messenger cascades, and transcriptional responses are essential to uncover the detailed biological functions of these hormones. Meanwhile, Future research should further investigate the precise biological mechanisms underlying the synergistic effects of gut-brain axis hormones. This review is subject to several limitations. First, findings across studies may vary due to differences in surgical techniques, such as Roux-en-Y gastric bypass versus sleeve gastrectomy, which elicit distinct hormonal responses. Second, heterogeneity in patient populations, including baseline metabolic status, age, sex, and genetic background, may affect hormonal dynamics and therapeutic outcomes. Third, most available studies have relatively short follow-up durations, limiting insight into long-term hormonal adaptation. Finally, confounding factors such as diet, physical activity, and medication use are not always adequately controlled, which may bias interpretations of hormonal effects.
